# A comparison of a multistate inpatient EHR database to the HCUP Nationwide Inpatient Sample

**DOI:** 10.1186/s12913-015-1025-7

**Published:** 2015-09-15

**Authors:** Jonathan P. DeShazo, Mark A. Hoffman

**Affiliations:** Department of Health Administration, School of Allied Health Professions, Virginia Commonwealth University, Grant House Room 201, 1008 East Clay Street, P.O. Box 980203, Richmond, VA 23298-0203 USA; Department of Biomedical and Health Informatics, School of Medicine, University of Missouri – Kansas City (UMKC), 2411 Holmes, MG-203B, Kansas City, MO 64108-2792 USA

## Abstract

**Background:**

The growing availability of electronic health records (EHRs) in the US could provide researchers with a more detailed and clinically relevant alternative to using claims-based data.

**Methods:**

In this study we compared a very large EHR database (Health Facts©) to a well-established population estimate (Nationwide Inpatient Sample). Weighted comparisons were made using t-value and relative difference over diagnoses and procedures for the year 2010.

**Results:**

The two databases have a similar distribution pattern across all data elements, with 24 of 50 data elements being statistically similar between the two data sources. In general, differences that were found are consistent across diagnosis and procedures categories and were specific to the psychiatric–behavioral and obstetrics–gynecology services areas.

**Conclusions:**

Large EHR databases have the potential to be a useful addition to health services researchers, although they require different analytic techniques compared to administrative databases; more research is needed to understand the differences.

## Background

Quality measurement and many health services research studies traditionally rely on administrative claims data. Discrepancies between medical claims-based data and clinical data within the patient medical record are well established [1–3], and claims data has been more consistently available than clinical data. However, recent advances in electronic health record (EHR) and health information exchange technology adoption may increase the availability of clinical data for research.

The Congressional Budget Office estimates that 90 % of physicians and 70 % of hospitals in the US will have electronic health records by 2019 [4]. Although electronic health records are primarily intended to support patient care, there is a high level of interest in using EHR data for secondary purposes such as biomedical and outcomes research, clinical process improvement, and epidemiological monitoring [5–8]. Despite some differences in EHR data quality compared to expert review and self-reported data [9], many health services researchers consider the EHR record to be more accurate compared to claims data and are calling for a shift away from claims-based measures to using clinical measures derived from the EHR [3, 10] for research, quality measurement, and performance monitoring. This transition away from claims data is demonstrated by the recent HITECH Act, which requires providers to report key clinical performance metrics directly from their EHR [11]. Limited information has been published on the benefits and limitations of using EHR data compared to claims-based data [12], yet it is critically important to understand these differences as we begin to rely on EHR data for research and payment in addition to supporting clinical care.

The potential advantages of using EHR data are numerous and are generally related to the detailed nature of the data as well as the benefits of being on a computer-based platform, yet the challenges posed are equally significant. The growing availability of electronic health records in the US could provide researchers with faster, less resource-intensive access to data, larger population samples, more data measurements, and additional types of data compared with primary data collection methods and claims data [6, 13]. However, researchers have noted significant challenges with using EHR data including privacy issues, variation in EHR data sources, inconsistent case definitions, poor data quality, and questionable representativeness of patient populations [8, 14, 15]. Many of these comparisons are based on public health or research data capture models, in which the data capture instrument and process were designed to capture comprehensive and analysis-ready data, while EHRs are generally implemented to provide clinically effective and rapid workflows with a higher tolerance for missing or absent data.

Notable platform-specific EHR databases that are frequently referenced in publications include the Veterans Health Administration databases [16, 17], the Kaiser Permanente Northern California Research Database [18], and the GE Centricity EMR Database [19]. Although valuable for many types of inquiry, using databases such as these to make population estimates may be problematic due to unique regional variations, inpatient or outpatient bias, or limited demographic population captured in the data.

A large EHR database, however, which is widely dispersed among different populations, hospital types, and care settings, may be used to make estimates of health at the population level or for broader groups such as the national universe of inpatients. Moreover, large EHR databases that contain detailed clinical data not found in claims databases could be very valuable to population health management if it were accurate, consistent, and representative of the population. As large EHR databases grow in numbers and in research utility, it becomes even more important to assess their strengths and limitations compared to existing data sources.

In this study, we compare a very large EHR database (Health Facts®) to a well-established population estimate (Nationwide Inpatient Sample). Although we are primarily concerned with the issue of representativeness, other considerations are relevant to our results. To assess representativeness, we calculate adjusted population estimates for encounter demographics, diagnoses, and procedures from both data sources, as well as the relative differences and t-values between the Nationwide Inpatient Sample and Health Facts® EHR populations.

### Cerner health facts®

Cerner Corporation, a leading EHR vendor, maintains one of the largest vendor-specific EHR databases, called Health Facts® (HF). Contributing organizations receive quality and benchmarking reports based on internal and external data. HF data is de-identified and HIPAA-compliant to protect both patient and organization identity. The patient-level data in HF includes encounter, medication, diagnosis, laboratory orders and results, pharmacy, and procedure values. These records are comprehensive and include over 300 data elements. For example encounter information includes details such as payer, discharge and admission sources, and care setting. Laboratory data includes collection sites, reference ranges, and the timing of collection and results. The database is longitudinal and hospital patients can be followed post discharge if they return to care settings within the same health system. At the time of this writing, HF contains data from more than 500 health care facilities across the United States representing 133 million encounters and 84 million patients over the past two decades. Health Facts® data is a publicly available resource from Cerner Corporation (Kansas City, MO).

### AHRQ HCUP Nationwide inpatient sample

The Agency for Healthcare Research and Quality (AHRQ) Healthcare Cost and Utilization Project (HCUP) Nationwide Inpatient Sample (NIS) is a valuable resource primarily intended for studies of inpatient hospital utilization and charges (http://www.hcup-us.ahrq.gov/nisoverview.jsp). It is the largest publicly available all-payer inpatient care database for the United States; in 2010, the NIS sampled approximately 20 % (1051) of all hospitals in 45 states. National estimates are based on a validated method of weights [20]. The NIS is released annually and contains standardized data elements reporting hospital characteristics, diagnosis and procedure codes, and other important data that can support research on medical treatment and effectiveness, quality of care and patient safety, impact of health policy changes, and use of hospital services within the United States. The research footprint and ultimate value of the HCUP NIS and other HCUP all-payer databases is significant, with thousands of studies publishing on HCUP data in peer-reviewed journals including hundreds of citations in top journals [21].

## Methods

### Data sources

Both the HF EHR database and the NIS data set capture patient-level data elements linked to inpatient encounters. Both data sources are available to the public at www.cerner.com/lifesciences and https://www.hcup-us.ahrq.gov/nisoverview.jsp respectively.

For this analysis we used an extract from the HF database made in January 2012. To make the HF data comparable to the 2010 NIS data set, we included only acute care discharges made during 2010 and excluded any stay longer than 365 days. We then created a weighting scheme similar to the weighting scheme used in the NIS database to make population estimates. First, we used 2010 American Hospital Association data stratified by region and hospital size to determine how many hospitals and discharges there were in the universe. We then assigned a weight to each hospital in HF that reflected its representation in the stratification. The formula for weight used is: (# in AHA universe/# in HF for each stratum). Strata used are region and bed size. For example, each 200–299 bed hospital in the Northeast in the HF sample is weighted to be equivalent to 6.23 hospitals in the universe. Because of limitations as a convenience sample, this weighting scheme is not balanced (i.e., we did not try to have the same size groupings) and does not take into account teaching hospitals as the NIS does. We used HCUP NIS data for 2010 for the comparison with no additional modifications.

### Analytic methods

Population estimates were calculated using SAS9 (SAS Institute Inc., Cary, NC, USA). Weighted estimates of diagnoses were aggregated using Major Diagnostic Category (MDC) groupings and included primary (i.e., first listed) diagnoses recorded for an encounter in the respective database. Procedures were aggregated according to high-level Clinical Classification Software (CCS) groupings and also included primary (first listed) procedure codes. ICD-9-CM code mappings for MDC and CCS groupings can be found on the HCUP website.

Standard errors were calculated and presented along with the NIS-weighted count estimates. HF provides complete enumeration of discharges from the source; therefore, no standard errors are presented. To compare the two data sets, we use relative difference and present the t-value. Relative difference is represented by the absolute value of (NISest-HFest)/NISest. The t-value is calculated as the difference between the estimations divided by the standard error (NIStot-HFtot)/(NISeststd err). A hypothesis-driven comparison of two data sets consisting of very large data sets such as these would result in statistical significance with very little difference. Therefore, we assess the significance of the t-value at 95 % confidence and, due to such large sample sizes, also at 99.9 % confidence. For those that are significantly different, we also provide the relative difference to assess the magnitude.

## Results

Encounters and demographics are presented in Table 1.

**Table 1:** Encounters and demographics: comparison of nationwide inpatient sample (NIS) and health facts® (HF) EHR population

	HCUP NIS		HF	Rel diff (%)	*t*-value
		Std. error			
Total discharges	39008298 (100.00 %)	723539	38787005	0.57	0.31
Northeast	7579674 (19.43 %)	317987	7603789 (19.60 %)	0.32	0.08
Midwest	8839256 (22.66 %)	320253	8897944 (22.94 %)	0.66	0.18
South	14985984 (38.42 %)	483009	15055887 (38.82 %)	0.47	0.14
West	7603384 (19.49 %)	294165	7229385 (18.64 %)	4.92	1.27
Length of Stay (Mean)	4.7	0	4.75		
Length of Stay (Median)	3	N/A	3		
Age (Mean)	48.36	0.37	47.38	2.03	2.65*
Male	16485595 (42.26 %)	312023	16207543 (41.79 %)	1.69	0.89
Female	22436406 (57.52 %)	429631	22549349 (58.14 %)	0.50	0.26
In-hospital Deaths	740748 (1.90 %)	17443 (0.03 %)	633121 (1.63 %)	14.53	6.17*

*Indicates *t*-values over 1.960. These are categories that are significantly different between the data sets at 95 % confidence

At the discharge level, the regional counts of discharges are generally comparable between the NIS data set and the HF EHR population. However, the HF population tends to be slightly younger (by approximately 1 year) and report fewer in-hospital deaths (1.63 vs. 1.90 %) compared to the NIS data set.

Tables 2 and 3 present comparisons on diagnoses and procedures, respectively.

**Table 2:** Diagnoses: comparison of nationwide inpatient sample (NIS) and health facts® (HF) EHR population

		Total number of discharges			Comparison		Percentage of total discharges	
	Major diagnostic category and name	HCUP		HF	Rel Diff (%)	*t*-value	HCUP	HF
	Diseases & disorders of:		Std. Error				%	%
1	Nervous System	2350764	58581	2373818	0.98	0.39	6.03	6.12
2	The Eye	56970	2792	52699	7.50	1.53	0.15	0.14
3	The Ear, Nose, Mouth & Throat	430252	15310	453087	5.31	1.49	1.1	1.17
4	The Respiratory System	3816282	72030	3993373	4.64	2.46*	9.79	10.29
5	The Circulatory System	5315827	133686	5752792	8.22	3.27*	13.64	14.82
6	The Digestive System	3473022	69682	3688959	6.22	3.10*	8.91	9.51
7	The Hepatobiliary System & Pancreas	1147155	27534	1175248	2.45	1.02	2.94	3.03
8	The Musculoskeletal System & Connective Tissue	3530758	96593	3161459	10.46	3.82*	9.06	8.15
9	The Skin, Subcutaneous Tissue & Breast	1007290	20998	1003193	0.41	0.20	2.58	2.58
10	Endocrine, Nutritional & Metabolic Diseases & Disorders	1269130	28332	1338294	5.45	2.44*	3.26	3.45
11	The Kidney & Urinary Tract	1684021	35948	1667673	0.97	0.45	4.32	4.3
12	The Male Reproductive System	193562	8343	212178	9.62	2.23	0.5	0.55
13	The Female Reproductive System	681145	19395	214863	68.46	24.04*	1.75	0.55
14	Pregnancy, Childbirth & The Puerperium	4323036	145371	1609355	62.77	18.67*	11.09	4.15
15	Newborns & Other Neonates With Condition Originating In Perinatal Period	4062084	135500	3121359	23.16	6.94*	10.42	8.04
16	Blood, Blood Forming Organs, & Immunological Disorders	531030	14971	526763	0.80	0.29	1.36	1.36
17	Myeloproliferative Diseases & Disorders, Poorly Differentiated Neoplasms	353805	27161	334898	5.34	0.70	0.91	0.86
18	Infectious & Parasitic Diseases, Systemic Or Unspecified Sites	1260596	29170	1111343	11.84	5.12*	3.23	2.85
19	Mental Diseases & Disorders	1517367	92216	862761	43.14	7.10*	3.89	2.22
20	Alcohol/Drug Use & Alcohol/Drug Induced Organic Mental Disorders	484570	39402	181993	62.44	7.68*	1.24	0.47
21	Injuries, Poisonings & Toxic Effects Of Drugs	609005	14033	560075	8.03	3.49*	1.56	1.44
22	Burns	42428	7177	46046	8.53	0.50	0.11	0.12
23	Factors Influencing Health Status & Other Contacts With Health Services	652276	31948	883098	35.39	7.22*	1.67	2.28
24	Multiple Significant Trauma	104919	8205	79620	24.11	3.08*	0.27	0.21
25	Human Immunodeficiency Virus Infections	77981	8095	49029	37.13	3.58*	0.2	0.13
	All Diagnosis	38975272		38808845				

*Indicates *t*-values over 1.960. These are diagnoses that are significantly different between the data sets at 95 % confidence

**Table 3:** Procedures: comparison of nationwide inpatient sample (NIS) and health facts® (HF) EHR population

		Total number of discharges					Percentage of total discharges	
	CCS Principal Procedure Category and Name	HCUP	Std. error	HF	Rel Diff (%)	*t*-value	HCUP	HF
							%	%
1	Operations on the nervous system	768496	42694	831098	8.15	1.47	3.12	2.80
2	Operations on the endocrine system	101141	7645	57033	43.61	5.77*	0.41	0.19
3	Operations on the eye	26432	2121	25783	2.46	0.31	0.11	0.09
4	Operations on the ear	15706	1335	25066	59.60	7.01*	0.06	0.08
5	Operations on the nose; mouth; and pharynx	133650	8828	127031	4.95	0.75	0.54	0.43
6	Operations on the respiratory system	659275	26693	749931	13.75	3.40*	2.68	2.52
7	Operations on the cardiovascular system	3459861	151346	4361495	26.06	5.96*	14.05	14.68
8	Operations on the hemic and lymphatic system	162493	11793	218612	34.54	4.76*	0.66	0.74
9	Operations on the digestive system	3316780	112496	3144045	5.21	1.54	13.47	10.58
10	Operations on the urinary system	540236	35296	599543	10.98	1.68	2.19	2.02
11	Operations on the male genital organs	1147705	51821	705496	38.53	8.53*	4.66	2.37
12	Operations on the female genital organs	700797	27975	793889	13.28	3.33*	2.85	2.67
13	Obstetrical procedures	3850461	173578	3986028	3.52	0.78	15.63	13.41
14	Operations on the musculoskeletal system	2958608	126795	2229428	24.65	5.75*	12.01	7.5
15	Procedures on the breast	760317	32677	717334	5.65	1.32	3.09	2.41
	Miscellaneous diagnostic and therapeutic procedures	6025241	556092	9597019	59.28	6.42*	24.47	32.3
	All Procedures	24627198	1369184	18837298				

*Indicates *t*-values over 1.960. These are procedures that are significantly different between the data sets at 95 % confidence

Nearly all of the encounters for both the NIS and HF had primary (i.e., first listed) diagnoses, equaling roughly 39 million encounters for each. In all but two of the MDC disease categories (14, 15), the percentage of the overall proportion of discharges in the NIS data set is within 2 % of the overall proportion of the HF population, indicating that the distribution of disease is very similar between the two data sources. Actual counts of encounters follow a similar pattern on comparability. With 95 % confidence in detecting a difference, 15 of the 25 MDC show statistically significant differences between the data sets. The relative difference is smaller than 5 % in one category, and smaller than 10 % in 5 categories. Although many MDC groups are consistent between the NIS data set and HF population, there are significant differences in the number of primary diagnoses in obstetrics and gynecological diagnoses (MDC 13, 14, 15), as well as psychiatric and behavioral diagnoses (MDC 19, 20).

About two-thirds of the NIS data set and one-half of the HF population had procedure codes associated with the encounter. Despite representing significantly fewer procedures overall, the proportions of procedures done in each CCS category in the HF EHR population were very similar to the proportions in the NIS data set. With 95 % confidence, 10 out of 16 NIS CCS categories are different between the data sets. However, only four of the procedure categories (9, 11, 13, 14) had proportions that differed by more than 2 % between the NIS data set and the HF population.

## Discussion

Regarding the demographic data on encounters, the comparison of the NIS data set with the HF EHR population shows strong similarities. The HF EHR population is slightly younger by about a year and reported slightly fewer in-hospital deaths. The small difference in age and mortality could possibly be explained through the distribution of hospitals in the HF database. Compared to the NIS data set, small hospitals (<99 beds) are overrepresented in the HF EHR database. If larger hospitals tend to have older patients and increased mortality compared to much smaller hospitals, then this may be reflected in the results. The weighting system applied to HF for this analysis is designed to adjust for much of this variation, yet some difference may remain.

Comparisons of the NIS data set and the HF population for diagnoses and procedures show mixed results. With the exception of a few diagnoses, MDC categories in the HF EHR population were consistent with the NIS data set. Psychiatry and behavioral (*Alcohol/Drug Use & Alcohol/Drug Induced Organic Mental Disorders, Mental Diseases & Disorders*) diagnoses as well as obstetrics and gynecology (*Diseases & Disorders Of The Female Reproductive System, Pregnancy, Childbirth & The Puerperium, Newborns & Other Neonates With Conditions Originating In Perinatal Period*) diagnoses are much less frequently represented in the HF EHR population compared to the NIS data set. It is possible that these differences could be attributed to selection bias of hospitals in the HF database resulting in hospitals that see fewer psychiatry and obstetrics and gynecology cases. For example, hospitals who adopted this specific EHR may see fewer of these kinds of cases. Another, more likely, explanation is that many of those services were still using either paper-based clinical records or a separate EHR system during the study period. This would result in fewer diagnoses in these categories within the hospital EHR. Mental health, substance abuse, and reproductive/sexual health are identified as are identified as “sensitive health information” in the recommendations of the National Committee on Vital and Health Statistics to the Secretary of Health and Human Services [22]. Some participating institutions may have compartmentalized this data from the less sensitive EHR data or embraced emerging security features of the EHR that allow patients to have greater control over the privacy of their health data. Information about the frequency of procedures was generally less consistent between the data sources compared to diagnoses. Although 11 of the 15 procedure categories in the NIS data set are proportionally within 2 % of the HF population, the HF population reports many fewer procedures overall compared with the NIS data set. Prior research has identified that EHR databases have tended to capture fewer data elements related to provider orders [23], which may explain the difference. Another possible explanation is that not all contributing sites are able to supply procedure codes to the database.

There are overarching considerations to the differences between EHR databases and claims-based samples identified in our findings. First and perhaps primarily, they are different resources created and predominantly used for distinctly different purposes. Prior research has shown claims coding can be markedly different than patient problems captured by providers [1, 24, 25]. Neither claims nor medical records are primarily collected for research so one is not necessarily more ‘correct’ than the other. Claims data in general has the advantage of years of validation and research use. Comparably, questions of internal validity and data integrity surround EHR data. Researchers are beginning to address many of these issues by developing innovative data infrastructure and study designs [14, 26]. In contrast, the HCUP NIS is a validated and trusted data source; however, it lacks much of the detailed clinical information that is available through large, longitudinal, detailed EHR databases.

Differences between claims-based data and EHR data found in our analyses may have implications for quality of care reporting since many federal measures are derived from claims data. Many federal-based quality reporting measures are based on claims data and the significance of the differences in reporting quality measures through the EHR are not well understood. For example, EHRs typically have more diagnosis fields (we found nearly 100 in this sample) compared to claims data, which may be significant when excluding cases from quality measures. Also, concepts are documented in claims data that are not documented in EHRs, and vice versa.

Additionally, EHR data may capture health care provided to patients yet not billed for that may not be reflected in the claims-based data, which could affect the comparison between the two data sets. The EHR sample included uninsured and self-pay patients, which the claims data do not have. Although EHRs may be more comprehensive in this regard, it complicates the comparison between the two. Finally, this analysis focused on in-patient hospitalizations; comparison of outpatient data might yield different results.

EHR data is a new type of data with new research possibilities, rather than being used as a supplement or substitute for claims based study designs. For example, EHR data may be preferred to claims data when clinical outcomes such as laboratory tests are required, or a level of clinical specificity is needed. EHR data may be preferred when the complete lists of diagnosis are needed, as many claims data will use only the first 25 or so diagnosis. Some data from the EHR such as problem lists, reflect clinician assigned diagnoses as they work the case, rather than codes assigned by a professional medical coder.

### Limitations

Some limitations of this study stem from the methodological differences between the NIS and HF data sets. NIS sampling is based on sampling techniques validated to represent hospitals across the country. Although the weighting scheme applied to HF is adjusted somewhat to reflect a national population of hospitals, it was not as detailed as the NIS weighting scheme and likely prone to error, particularly in the western region of the US where fewer hospitals contribute to the HF database. These data were collected through a single EHR vendor and it is unknown how they may differ (if in any way) from data obtained elsewhere. In addition, there may be differences between the data sets in the primary diagnosis criteria and definition. There may also be differences due to the inclusion of self-pay and uninsured patients in the EHR data. It is also possible that there were structural or service line differences between the hospitals in each sample. For example, some hospitals may not have Obstetrics or Psychiatry. Significant differences in either of these may skew the results.

A second limitation of the study reflects the transition from paper-based records to EHRs occurring during the study period. It is very likely that in the study year (2010) many of the hospitals contributing to the HF EHR database were not completely EHR-based throughout the hospital, potentially resulting in missing information within the EHR database. More research is needed to compare EHR populations to claims samples to detect changes in these differences over time.

A third important limitation of the study is that the analysis was done on hospital data and not outpatient data. Inpatient and outpatient EHR adoption rates varies, as does the documentation and coding practices. Prior research has found variation between outpatient EHR data and Medicaid claims [27], but promising methods for applying claims-based quality measures to outpatient EHRs are being developed [28]. More research is needed into understanding the developing role of outpatient EHR data in health services research and quality measurement.

As already described, HF is not a random sample and the EHR population at the time of this analysis consisted of proportionately more small (<99 bed) hospitals compared with the NIS, with a greater concentration of hospitals in the northeast region. Addressing these differences required an unbalanced weighting scheme with weights that varied by a factor of 100. Also, there may be differences in health care organizations that choose this specific EHR vendor compared to those who choose another vendor.

## Conclusions

This study compared demographic variables, diagnosis groups, and procedure categories in the HCUP NIS data set and HF EHR population. Compared to the NIS, the HF EHR population is slightly younger with lower in-hospital mortality. There tends to be about the same distribution of principal diagnoses between the groups, with the HF EHR sample reporting fewer psychiatry, behavioral, obstetrics, and gynecology diagnoses. Compared to the NIS data set, HF captured fewer procedures overall, and also in relation to surgeries involving male and female genital organs and obstetric procedures.

These findings improve our understanding of the differences between established claims-based databases and large EHR databases and support evidence that large EHR databases have the potential to be a useful addition to health services researchers.

More research is needed to understand the internal and external validity of large EHR databases as it pertains to population health research, specifically in the interpretation of results from EHR databases. There is also a significant need for the development of innovative methodologies that may be required to fully utilize these rapidly growing data sets.

## Appendix

**Table 4:** Weighting scheme

Region	Bed Size	# of Hospitals in HF Sample	# of Discharges in Sample (Health Facts)	# of Discharges in Universe (AHA)	Discharge Weights (universe/sample)	# of HF Discharges with Dx and Procs	Dx and Proc Weights (universe/sample)
Midwest	<99	28	13711	1226395	89.45	11197	109.53
Midwest	100-199	11	49244	1503133	30.52	18544	81.06
Midwest	200-299	5	51911	1644218	31.67	24916	65.99
Midwest	300+	10	152916	4526492	29.60	59741	75.77
Northeast	<99	23	131065	385176	2.94	30687	12.55
Northeast	100-199	7	44618	1087590	24.38	35543	30.60
Northeast	200-299	14	230950	1437848	6.23	74904	19.20
Northeast	300-499	6	94831	2021580	21.32	48317	41.84
Northeast	500+	7	243512	2689519	11.04	87817	30.63
South	<99	29	60081	1632606	27.17	21043	77.58
South	100-199	8	69961	2685597	38.39	26535	101.21
South	200-299	9	116086	2543060	21.91	56292	45.18
South	300-499	6	85349	3766138	44.13	22595	166.68
South	500+	6	269477	4433500	16.45	109494	40.49
West	<99	10	21522	778899	36.19	8466	92.00
West	100-199	5	32114	1603107	49.92	2379	673.86
West	200-299	4	46321	1525325	32.93	13427	113.60
West	300+	3	80072	3325308	41.53	22570	147.33

## Abbreviations

AHRQ: Agency for healthcare research and quality
CCS: Clinical classifications software (grouping)
EHR: Electronic health record
HCUP: Healthcare cost and utilization project
HF: Cerner health facts database
HIPAA: Health insurance portability and accountability act
HITECH: Health information technology for economic and clinical health act
MDC: Major diagnostic category
NIS: National inpatient sample
